# Understanding perspectives on smoking cessation based on Self-Determination Theory: A qualitative study

**DOI:** 10.18332/tpc/211451

**Published:** 2025-12-23

**Authors:** Maarit Malin, Minna Majuri, Ville Lahtinen, Kari Reijula

**Affiliations:** 1 Department of Public Health, Faculty of Medicine, Helsinki University, Helsinki, Finland; 2 Helsinki University Central Hospital, Helsinki, Finland; 3 Haaga-Helia University of Applied Sciences, Helsinki, Finland

**Keywords:** smoking cessation treatment and support, Self-Determination Theory, qualitative research, individuals’ perspectives, healthcare professionals

## Abstract

**INTRODUCTION:**

Implementing and maintaining smoking cessation requires high motivation. This study examines the role of Self-Determination Theory (SDT) in enhancing smoking cessation strategies by highlighting the significance of psychological needs for lasting behavioral change. We aimed to explore how participants perceive the importance of SDT's basic psychological needs – autonomy, competence, and relatedness – in the context of smoking cessation treatment and support (SCTS).

**METHODS:**

This qualitative study, employing SDT-driven content analysis, utilized a co-design workshop held in Finland in 2019, where 12 participants engaged in group dialogues. The workshop dialogues were examined to determine whether SDT influenced participants’ responses regarding SCTS.

**RESULTS:**

The qualitative analysis emphasized the respondents' need for autonomous motivation. The study highlighted the importance of acknowledging individual differences and psychological needs for effective support in SCTS. We identified ten categories: Addiction, Aids and appliances, Characteristics, Collective quitting, Expert advice, Follow-up data, Personal experience, Rewards, Health political decision-making, Ways of quitting smoking. All were linked to basic psychological need. The desire for autonomy was the most common theme (41.2%) followed by competence (34.6%) and relatedness (24.2 %).

**CONCLUSIONS:**

Health practitioners should recognize and address the basic psychological needs of autonomy, competence, and relatedness when assisting smokers in their cessation efforts. By offering meaningful choices to support autonomy and enhance competence, and by providing positive, informative feedback while empathizing with the smoker’s situation, professionals can improve the effectiveness of SCTS interventions. To be truly effective, support must be tailored to individual differences and needs.

## INTRODUCTION

Smoking is the top behavioral risk factor for mortality worldwide, and was responsible for over 175 million deaths and nearly 4.30 billion years of life lost between 1990 and 2021^1^. In 2019, smoking tobacco was the leading risk factor for death among males^2^. The total number of global smokers continues to rise, although the rate has declined in many developed countries in the past ten years^2^. More than 1 billion people smoked tobacco regularly in 2019, and almost 8 million deaths were attributed to smoking^2^. The prevalence of smoking varied substantially across countries^2^. Around 80% of the world’s tobacco users live in low- and middle-income countries^3^. The global age-standardized smoking prevalence was estimated to be 28.5% among males and 5.96% among females in 2022^1^. In 2022, about 11% of Finns aged 20–64 years smoked daily, and smoking among men has increased slightly^4^. The tobacco epidemic has many outcomes which lead to health risks, deaths and rising economic costs^3^.

Smoking cessation, treatment and support (SCTS) is the single most effective intervention to reduce the risk of premature disease, disability and death^5^. Its support of smokers’ psychological needs and intrinsic motivations has demonstrated clinical benefits^6^. Despite the increasing number of mobile health technologies designed to help smokers quit^7^, only a few have been scientifically validated^8^, and many are of low quality^9^. In Finland, there have not been any SDT-based gamified mCessation solutions that complement the current SCTS. Our preliminary understanding was that the basic psychological needs of autonomy, competence, and relatedness might be reflected in the answers.

**Figure 1:**
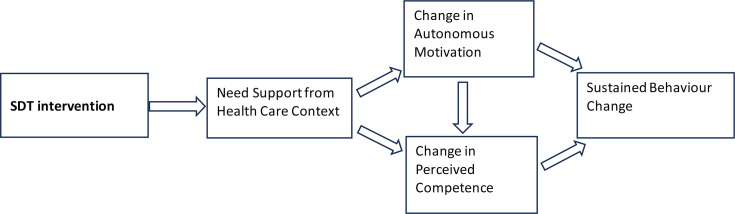
Core elements of SDT process model of health-promoting behavior change^13^

The SDT is increasingly used as a theoretical framework for health promotion and fostering health behaviors^10-12^, including abstaining from tobacco use^13^. Taking autonomy, competence and relatedness into account is crucial for understanding SCTS^13^. SDT-informed interventions for tobacco dependence have demonstrated cost-effectiveness^14^. Individuals feel autonomous when their actions are driven by a sense of choice and volition, but they feel controlled when they experience external pressure or coercion to think, feel or act in specific ways^15^. A person with a greater need for autonomy may develop more self-determined motivation to quit smoking than someone with a less need for autonomy, who in turn might prefer an expert telling them how best to quit^15,16^. For instance, smokers are autonomously self-regulated (ASR) if they try to quit smoking because it holds personal significance for them (identified regulation) or aligns with their deeply held values and aspirations (integrated regulation). Conversely, smokers are controlled if they attempt to quit due to pressure from a healthcare provider or spouse (external regulation) or because they feel compelled by guilt or shame (introjected regulation). According to SDT, only individuals who feel they are making a change willingly (autonomously) are likely to gain from support that enhances their competence^17^: they are more fully committed, persistent and effective than when their motivations are externally controlled^18^. For instance, a smoker might be motivated to quit due to incentives from their employer, such as extra pay, or they might want to understand their nicotine addiction and appreciate the benefits of quitting, such as better work performance, participation in sports, or a meaningful hobby. In these instances, although the intensity of motivation may remain constant, the type and direction of the motivation clearly differ^18^. Figure 1 describes how supporting autonomy through treatment increases both autonomous motivation and perceived competence for health behavior change^13,19^. Our objective was to understand individuals’ experiences and view of various SCTS methods including mCessation. In our pilot study we evaluated how these methods align with the SDT and which of the basic psychological needs of autonomy, competence and relatedness were the most important for SCTS.

## METHODS

### Study design, setting and ethics

We used an exploratory qualitative study design, and reporting guided by the Standards for Reporting Qualitative Research (SRQR)^20^. Our qualitative methods design involved analyzing data using content analysis based on the SDT. The qualitative data were collected in a workshop held in April 2019. MaM and VL planned, organized and conducted the workshop, and we used a consumer-driven approach to collect the qualitative data. The aim of the workshop was to gather insights from respondents regarding their perceptions of available smoking cessation support methods, and to assess whether we have successfully incorporated key SDT-based elements into our developed mCessation application. The results of the pilot study are intended to be used for further development of the application. The cross-disciplinary research team was made up of occupational health experts. The researchers have influenced the study in the following ways: the co-design workshop follows the principles of social marketing, and the SCTS interpretation adheres to the standards of medicine and health sciences. First, we asked the participants to independently identify what are the three most important and three least important features of both SCTS and mCessation. Next, they brainstormed and proposed new ideas and approaches for SCTS and mCessation.

The participants received visual cards detailing the various SCTS methods available in Finland and the features of the mCessation program we had developed. The cards included following pictures: smoking cessation tools comprised nicotine replacement therapy, professional support from healthcare providers, smoking-cessation medicines, cessation groups, phone-based psyche education, online psychoeducation online peer support groups^21^; nicotine-free environments^22^; mCessation apps^7,23,24^ ; text message health apps^25^; and hypnotism, websites, smoking-cessation guidebooks, and acupuncture. The list also included methods used by smokers for which there is no strong scientific evidence. We carefully informed of the study about the researcher’ backgrounds before the session. They signed their consent for audio recordings, which were parts of the co-design sessions. We recorded the group discussions, and we subsequently transcribed the de-identified data.

### Participants

**Figure 2:**
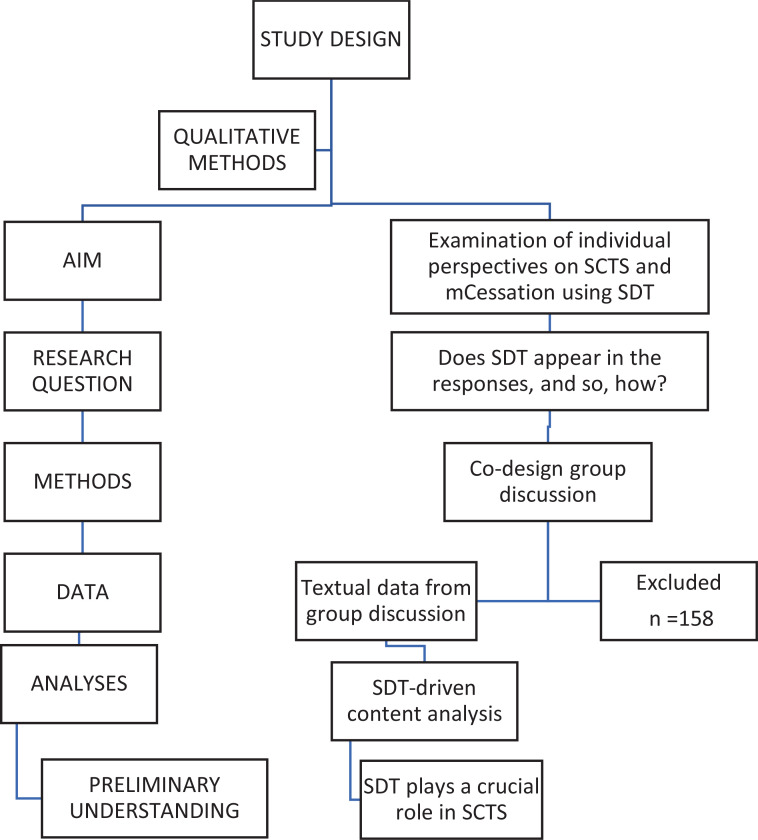
Study flow: design of a 2019 qualitative study to assess 12 individual perspectives on a workshop based on Self-Determination Theory (SDT) in Finland

The participants consisted of 12 students recruited from the collaborating Business College Helsinki (Table 1). The school was selected because the respondents need to have an interest in and contribute to the co-development of both SCTS and mCessation. The sample was purposive selected from among 170 adult students with certain expertise of the Information and Communications Technology degree program. Two investigators (MaM and VL) collaborated with the head teacher (HH) and teacher (TT). They planned the co-design workshop together, including the exclusion and inclusion criteria of the study. One former smoker used nicotine replacement therapy (NRT). We excluded students who were aged <18 years. The inclusion criteria required that participants have an interest in SCTS and mCessation, indicating that, for one reason or another, they had a personal motivation to quit smoking or test and use mCessation. We did not exclude non-smokers because the essential purpose of the workshop was to assess mCessation and gather information for further research. We assumed that it could positively affect the achievement of saturation. Thus, the participants needed to have a personal interest in the digital applications, and they were selected from the program mentioned earlier (Figure 2).

**Table 1:** Background characteristics of the respondents, Finland, 2019 (N=12)

*Characteristics*	*Male* *n*	*Female* *n*
**Total**	8	4
**Age** (years), range	18–47	20–40
**Smoking status**		
Casual	1	0
Current	1	3
Former	2	0
Non-smoker	4	1
**NRT**	1	0

NRT: nicotine replacement therapy.

### Qualitative analyses

The qualitative theory-driven content analyses of the co-design materials were conducted by MaM and MiM. To enhance transferability, the researcher ensured triangulation of the data and achieved theoretical saturation. In cases of coding disagreements, discussions were held to achieve a consensus, involving at least two researchers as necessary. Researchers approached the recorded group session data from the perspective of SDT-driven content analysis^26^. They adhered to the principles of deductive content analyses^27^, beginning by analyzing whether and how the respondents expressed the core elements of the SDT. The analysis frame consisted of autonomous and controlled motivation and three main psychological needs: autonomy, competence and relatedness to intrinsic motivation. These concepts are also called the main categories in this article. We read the transcribed data several times, and MaM derived codes using ATLAS.ti software. Every sentence was repeatedly analyzed by two investigators and clustering resulted in 133 subcategories (Table 2).

**Table 2:** Results of theory-driven content analyses of workshop textual data, consisting of 3 SDT-based main categories and 10 upper categories with 133 subcategories

*Main categories*	*Upper categories*	*Subcategories*	
**Autonomy and competence**	Addiction	Craving for nicotine	Relapse
		Craving for tobacco	Snuff
		Desire	Paying attention to smoking
		Nicotinism	Thinking about smoking
**Autonomy**	Aids and appliances	Acupuncture	Hypnosis
		Alternative physical activity	Massage
		Casual actions	Medication
		Exercise	Personal messages
		Handbook	Psychoeducation
		Help	Psychoeducation after relapse
		Relaxing and meditation	Reminders
		Replacement therapy	Focusing attention on smoking
		Weaning off tobacco	Western medicine
**Autonomy, competence relatedness**	Characteristics	Captivating game	Digital support
		Affordable	Distance
		Amusement	Expensive
		Availability	Games
		Concrete	Impractical
		Digital platform	Innovative
		Interactive	Personal matter
		Practicality	Reactionary
		Safety	Self-evident
		Usefulness	
**Relatedness, competence**	Collective quitting	Acting in a group	Peer pressure
		Community spirit	Peer support
		Competition	Reference group
		Internet peer support	Indifference
**Competence**	Expert advice	External help	Professional advice
		Process of quitting	Professional orientation
**Autonomy**	Follow-up data	Awareness of own progress	Information about experiences
		Continuity	Information about goals
		Control	Information about health, present state
		Health data and progress	Information about own action
		Influence on health	Information about quality of life
		Time usage information	Life expectancy
**Autonomy, competence**	Personal experience	Contradictions	Acknowledging feelings
		Equality	Reducing stress
		Failure	Sense
		Own experience	Usual manner
		Pain	Time management
		Private matters	
**Competence, relatedness**	Rewards	Online rewards from games	Team reward offline
		Personal award offline	Team reward online
		Personal award online	Workplace reward for quitting
		Personal reward	Public reward for quitting
		Team reward	Rewards
**Competence**	Health political decision-making	Allocated help for young people	Nicotine-free environment
		Employee incentives	Oriental medicine
		General awareness	Scientific evidence-based information
		Harm of smoking	Smoking ban
		Information on the harm of smoking	Making it more difficult to smoke
		Tobacco tax	
**Relatedness**	Way of quitting smoking	Trying something new	Quitting as an anonymous person
		Experiment in quitting	Quitting in a team
		Disruption	Quitting seriously
		Goal orientation	Seeking help (from a specialist)
		Process of quitting	Quitting smoking
		Quitting as an individual	Reducing smoking
**Controlled motivation or amotivation**		Bypassing the information	External motivation
		Indifference	The rebellion against bans
**Autonomous motivation**		Self-direction	Encouragement
		Autonomy	Interest
		Perseverance	Efficacy
		Feedback	Empowerment
		Commitment	Being motivated
		Positivity	Internal motivation
		Motivation in leisure time	Success
		Quitting smoking	

Analysis of the data revealed that the respondents expressed different motivation types. We sorted these into main categories amotivation, controlled motivation and autonomous motivation. Next, we grouped the remaining ten upper codes into main categories (i.e. competence, autonomy and relatedness). The following step describes how motivation appeared in ten upper categories and how they were linked by the three main psychological needs: autonomy, motivation and relatedness (Figure 1). At no stage of the study did the participants receive any information on the SDT.

## RESULTS

**Figure 3:**
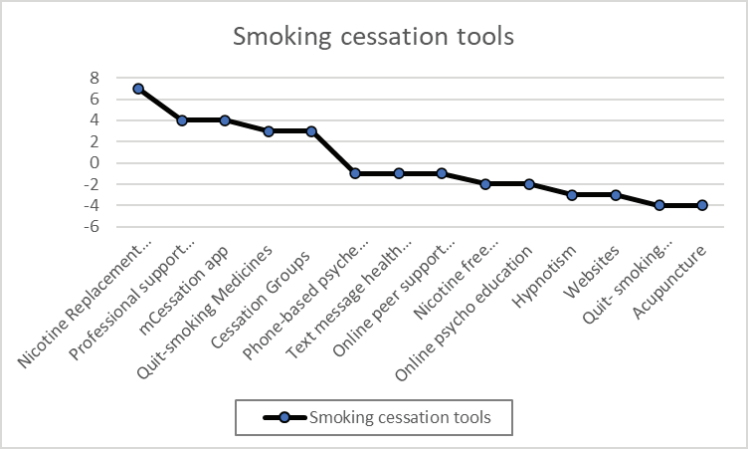
In the co-design workshop, participants assessed various smoking cessation methods in order of priority

Participants (n=12) ranked smoking cessation tool features in order of importance: nicotine replacement therapy, professional support from healthcare providers, and mCessation. Smoking cessation guidebooks were deemed least effective, as was acupuncture. Figure 3 displays the rankings of helpful and less helpful features. The vertical axis represents the value of different phases: nicotine replacement therapy, professional support from healthcare providers, mCessation apps, smoking-cessation medicines, cessation groups, phone-based psyche education, text message health apps, online peer support groups, nicotine-free environments, online psychoeducation, hypnotism, websites, smoking-cessation guidebooks, acupuncture. The summary shows features deemed most and least useful; a negative result indicates perceived less utility rather than harm.

### Qualitative results

We assessed how the participants perceived the SCTS and mCessation based on the SDT. In the workshop, we looked for ways in which to include features in and SCTS and the mCessation. The participants identified the features they considered important for supporting SCTS. These were then categorized into ten upper categories, all of which were associated with the basic psychological needs outlined by the SDT. Table 2 shows the SDT-based main categories (basic psychological needs: autonomy, competence and relatedness) and the upper categories and subcategories (n=133) that the content analysis of the workshop material revealed. The ten upper categories linked to the three basic psychological needs were: 1) Addiction; 2) Aids and appliances; 3) Characteristics; 4) Collective quitting; 5) Expert advice; 6) Follow-up data; 7) Personal experience; 8) Rewards; 9) Health political decision-making; and 10) Way of quitting smoking.

### Amotivation, controlled motivation and autonomous motivation

The respondents used several names for the different motivation types. We sorted the replies into the following codes: autonomy, being motivated, commitment, efficacy, encouragement, empowerment, feedback, internal motivation, motivation in leisure time, perseverance, positivity, self-direction, success and quitting smoking. We identified these as being connected to autonomous motivation, which stems from intrinsic motivation. In contrast, the following codes: external motivation, interest, indifference, bypassing information and rebellion against bans were connected to amotivation or controlled motivation. The respondents mostly emphasized autonomous motivation, which received 64 comments in total, whereas amotivation or controlled motivation only received 12 comments.

The participants reflected on the different types of motivation, and we found different levels of controlled motivation and amotivation. Some participants described how teenagers could be totally amotivated by smoking bans:

*‘Among teenagers, who want to rebel, if smoking is forbidden, I suppose, it may increase.’*

Saving money concerns external motivation, depending on what kind of value money has in one’s life. When external values are introduced and identified, internal motivation for quitting may be found and integrated:

*‘Seeing these results and how much money has been saved and so on … this helped me find some motivation …’*

The participants reflected that no one will read a long information list about smoking. The smoking quitter’s comment could be interpreted as a belief that information-based websites offer hardly any benefits to the smoking quitter. Information requires autonomy-orientation, which means a person taking an interest and orientating toward their environment by treating it as a source of relevant information:

*‘With information on websites, it can just be a long list of information. Nobody will read it.’*

The participants mentioned controlled motivation in different ways, but they mostly expressed autonomous motivation, when efficacy appears in different ways. Internalization and integration are facilitated by supportive social contexts that foster a sense of connection; thus, they enable feelings of efficacy and autonomy:

*‘Quitting in a team for the same reasons. You get clear feedback. I’m trying, and I can do it.’*

They highlighted their own motivation; that quitting is impossible without internal motivation. External pressure may push a person to quit, but it must have individual significance, which could be found in feelings of better health. Indeed, when smoking quitters have truly accepted the value of quitting as being personally important, they are more likely to follow through with this important behavior:

*‘It’s quite clear that if you get all kinds of health benefits, you’ll be more motivated to quit.’*

### Autonomy, competence and relatedness

Figure 3 illustrates the key elements identified by the participants as essential for supporting SC. We grouped these elements into ten main categories, all of which are strongly linked to the SDT. When categorizing the ten upper categories according to the SDT and basic psychological needs, we always returned to the authentic replies and the SDT literature. We quantified the importance of the ten main categories by counting the number of times each category was mentioned.

**Figure 4:**
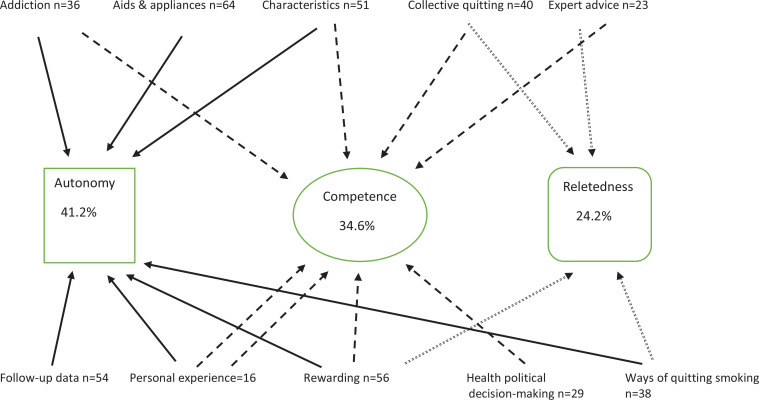
Results of content analysis of textual data from workshop. Number of ideas gathered from Finnish workshop held in April 2019 (N=12). The dashed arrows represent the autonomy, the solid arrows indicate competence, and the dotted arrows signify relatedness

The SDT-based content analysis (Figure 4) revealed that SCTS needs to consider humans’ basic psychological needs of autonomy, competence and relatedness. The replies revealed that people who are trying to quit smoking need an enhanced sense of autonomy. The feeling of competence manifested in different ways. In particular, the ‘health data and advancement’ and ‘different rewards’ codes included elements that could motivate individuals to continue not smoking and thus enhance their sense of competence. Collective quitting may play an important role in experiencing success as a group.

Competence can increase from the macro to the micro level. At the macro level, society can support people with nicotine addiction through legislation, such as requiring tobacco products to be kept out of sight in shops. At the micro level, employers can help individuals trying to quit by creating a nicotine-free work environment. The respondents highlighted the impact of political decisions on health and the role of employers in supporting these efforts. Smoking bans received mixed opinions. Although they make smoking more difficult, nicotine addiction may lead smokers to seek alternative solutions, such as using snus. Some respondents believed that quitting smoking was easier when smoking was made difficult and uncomfortable. They suggested finding alternatives such as distracting oneself with activities like playing games or exercising to help forget their craving. They concluded that some levels of external pressure (i.e. controlled motivation) may have both benefits and drawbacks in terms of successfully quitting smoking:

*‘A smoke-free environment, well it doesn’t always work, because, for example, if you’re not able to smoke, you can use snus or go and smoke somewhere else.’*

Autonomy is particularly evident when a smoker makes the decision to quit, how they will do it, and what kind of aids and support they will choose. Rewards are mostly associated with group quitting, when members can support and encourage each other. As regards expert advice, autonomy support was not very clear; it only became apparent in healthcare advice regarding what to do to successfully quit smoking:

*‘These professionals, they know best how to give instructions to quit smoking, and the smoking process, in which order you should start them.’*

Expert advice was seen as authoritarian, someone telling you what to do. Some respondents considered phoning a healthcare adviser and asking for help to quit smoking very old fashioned. Others appreciated the importance of contacting healthcare experts to receive support and help.

The importance of relatedness manifested in various ways. Quitters were able to support and learn from each other through shared experiences:

*‘Then there’s this support group, so you can talk to different people who might have similar problems and share all kinds of experiences and tips on how to get over it, and this mutual understanding, like helping other people get over it too.’*

*‘Then there’s this support group, it’s good ‘cause you can talk face to face with others who have the same problem, and like, as a group, you can get off nicotine together.’*

Some emphasized the significance of face-to-face meetings, while others highlighted the sense of togetherness and being able to identify with people facing the same challenge, nicotine addiction:

*‘Quitting in a team because you can learn from other smoking quitters’ achievements and mistakes.’*

The participants stressed the importance of common goals, regardless of the quitting method. This is particularly crucial if workplace teams can earn rewards for successfully quitting smoking. Those who used a digital platform agreed that the platform should include social elements, especially if it incorporated gamification features. The respondents’ answers were highly individualized, regardless of whether they were smokers or users of nicotine products. Consequently, the responses from smokers did not differ from those of non-users of nicotine products.

## DISCUSSION

According to the respondents, successful smoking cessation is most likely when the person quitting has autonomous motivation. External pressure alone is not enough for a lasting change. The three basic psychological needs of autonomy, competence, and relatedness were prominently reflected in the responses across ten upper categories. Of these, the need for autonomy appeared the most frequently.

The respondents emphasized autonomous motivation, which arises from internal drive. In practice, successful, lasting smoking cessation is impossible without internal motivation. Behavioral patterns change require patient motivation, with the SDT suggesting that autonomous motivation is most effective^13^. Teachable moments provided by health practitioners may elicit emotional responses, often distress^28^. Most individuals are already aware that smoking is harmful^28^. Our results are in line with those of previous studies: when an intervention succeeds in increasing autonomous motivation, it leads to a change in health behavior^13,19^. The respondents identified various types of motivation, emphasizing that autonomous motivation is crucial for successful smoking cessation. They also acknowledged that controlled motivation, such as the financial benefits of quitting, can be valuable for individuals attempting to quit^18^. Smoking bans can make quitting more difficult^22^. Certain age groups may rebel against such restrictions, which can undermine their motivation to quit^18^. Ideally, autonomous and controlled motivation complement each other. External pressure can initiate the quitting process and lead to individuals discovering internal motivation.

The respondents believed that SC support on websites is unnecessary because people generally do not read such information. This perspective aligns with the Causality Orientation Theory, a sub-theory of the SDT^13^, according to which individuals exhibit unique differences and needs. Engaging with websites in a self-regulated manner requires an orientation towards autonomy, which implies that autonomy-orientated individuals are inherently interested and thus more self-regulating^13,29^. Individuals with a strong autonomy orientation typically display prominent levels of intrinsic motivation. Those with controlled orientation are more focused on external contingencies and controls, resulting in lower levels of intrinsic motivation^13^. Finally, an impersonal orientation reflects the extent to which individuals perceive obstacles to achieving their goals, frequently experiencing anxiety and feelings of incompetence, and therefore more likely to be amotivated^18^. The impersonal causality orientation underpins a diffuse-avoidant identity style^13^. This raises a pertinent question: ‘What proportion of smokers exhibit a controlled or impersonal orientation, which is more predictive of poor well-being than an autonomy orientation?’. The concept of causality orientation provides valuable insights into why individuals exhibit varying levels of health, effectiveness and happiness, even within the same social context^13^.

The results correspond with the SDT: healthcare experts being autonomy-supportive predicts patients’ autonomous motivation, which in turn predicts changes in their sense of competence regarding quitting^19^. According to the SDT, self-determined motivation mostly stems from one’s own values or interest^13,15^. In the present study, this means quitting smoking because it has personal value and it satisfies the need for autonomy. In our study, some responses suggested that health professionals came across as authoritarian, deciding what the best method for quitting should be. This way of helping does not increase supportive autonomy among those attempting to quit smoking. Experienced healthcare professionals could increase patients’ feelings of competence. Competence can be supported on many levels: sound political decision-making enables individuals to succeed. The term competence is related to the concept of self-efficacy. The concept of perceived competence (PC) closely resembles that of self-efficacy, but they have distinct meanings. SDT focuses more broadly on motivation and needs, whereas self-efficacy according to Bandura^30^ emphasizes individuals’ beliefs in their capabilities^31^. Healthcare professionals should support the smoking quitter’s autonomy or self-determination, and enhance their sense of competence or belief in their own abilities^16,19,32^.

The need for relatedness manifested in various forms, and connections to nurses and other healthcare professionals were deemed crucial. This observation aligns with those of prior studies^33^. Although the individual’s sense of autonomy is vital in relatedness, it seems that healthcare professionals are not always perceived as autonomy supportive. This perception may be rooted in the individual’s personal experiences. The responses indicated that professionals recognize the importance of tailoring solutions to the individual needs of those attempting to quit smoking.

The respondents also emphasized the importance of peer groups. We identified both extrinsic and intrinsic goals among the respondents. If the same team receives rewards from their employer, their motivation tends to be more extrinsic than intrinsic. The respondents also underscored the significance of psychological support derived from relatedness. Social support from one’s environment can be crucial, and even non-smokers expressed interest in cessation support. Achieving these extrinsic goals together, such as receiving rewards, may lead to competence satisfaction and foster intrinsic goals, which in turn are associated with autonomous motivation^13,18^. In other studies using the COM-B Model, the importance of encouragement from family members is emphasized^34^.

The respondents assessed mCessation most important tool besides nicotine replacement therapy and psyche education of nurses. This aligns with other studies: mCessation has the potential to complement and enhance existing interventions, but its scientifically based development requires a better understanding of users’ needs^8^.

### Strengths and limitations

The study participants were not provided with any information about the SDT while they were evaluating the smoking cessation tools, to ensure that their answers were not influenced by the researchers. The recruitment and selection process were based on the respondents’ personal interest in SCTS. We used consumer-driven co-design methods, which are recommended for optimizing the user viewpoint. The respondents’ perspectives began to repeat, indicating that data saturation had been reached^35^. Although the number of participants was not very high, the data they produced were representative. This study has some limitations. Selection bias may have been possible among the participants, as the task of disseminating information at the school may have attracted a specific profile. The participants’ ages varied widely, as did their tobacco status. However, the responses from non-smokers did not differ from those of smokers, likely because the issue holds some relevance for them as well. This may affect the transferability of our findings to other contexts, such as evaluating professionals’ methods of implementing SCTS. Elitist bias is also possible, which is when a researcher selects only the most interesting comments; or holistic bias, which happens when a researcher overemphasizes an individual comment. A response bias is also possible, which means that respondents might be influenced by various factors and may not provide true, accurate, or valid answers^26,36^. More studies are needed regarding smoking cessation practices. The Treatment Self-Regulation Questionnaire (TSRQ), for example, should be used if we would like to gather more information and reasons for quitting^17,36^.

## CONCLUSIONS

By offering meaningful choices, i.e. supporting autonomy, which in turn enhances the feeling of competence, and providing positive and informative feedback, and empathizing with the smoker’s situation, professionals can enhance the effectiveness of SC interventions. Ensuring that SCTS are accessible to all smokers, regardless of their residence or socio-economic status, is crucial. Ultimately, if support is to be effective, it is crucial that individual differences and needs are acknowledged.

## Data Availability

Data sharing is not applicable to this article as no new data were created.
